# Optimizing Gait Outcomes in Parkinson’s Disease: The Effects of Musical Groove and Familiarity

**DOI:** 10.3390/brainsci15090901

**Published:** 2025-08-22

**Authors:** Emily A. Ready, Jeffrey D. Holmes, Eryn P. Lonnee, Jessica A. Grahn

**Affiliations:** 1 Department of Psychology, Western University, London, ON N6A 3K7, Canada; eready2@uwo.ca; 2 Centre for Brain and Mind, Western University, London, ON N6A 3K7, Canada; elonnee@uwo.ca; 3 School of Occupational Therapy, Western University, London, ON N6A 3K7, Canada; jholme@uwo.ca

**Keywords:** gait, Parkinson’s disease, music, rhythmic auditory stimulation

## Abstract

Background. Parkinson’s Disease (PD) is a neurological condition that can severely impair gait, often through changes to gait parameters including stride length, velocity, and variability. Therapeutic interventions such as Rhythmic Auditory Stimulation (RAS^®^) target gait dysfunction in PD by using the regular beat of music or metronome clips to cue normalized walking patterns. Previous research has suggested that auditory cue properties (e.g., familiarity and groove) and individual factors (e.g., beat perception ability and susceptibility to dual-task interference) influence auditory cueing treatment efficacy in healthy young and older adults; however, optimization of rhythmic cueing across individuals with PD remains understudied. Methods. To address this, we explored the effects of familiarity, groove, beat perception ability, and synchronization instructions on gait in patients with PD during accelerated auditory cues. Individuals with idiopathic PD were randomized to walk freely or synchronized to music and metronome cues played 10% faster than their baseline walking cadence. Musical stimuli varied in self-reported familiarity and perceived groove and beat perception ability was assessed to classify participants as good or poor beat perceivers. Results. Overall, high-groove music and synchronized walking elicited faster gait patterns compared to low-groove music and free walking, respectively, as demonstrated by increased gait velocity and cadence. Familiarity and beat perception ability did not significantly affect gait in individuals with PD. Discussion. Altogether, our results indicate that high-groove music and synchronized walking lead to the greatest gait improvements during cueing, regardless of beat perception ability. Conclusion. Future studies and clinical interventions should consider stimulus type and synchronization instructions when implementing cueing therapies for gait dysfunction in PD in order to optimize treatment responses.

## 1. Introduction

Parkinson’s Disease (PD) is a neurodegenerative movement disorder that severely impairs voluntary and controlled motor functions, such as walking [1]. Gait dysfunction in PD is characterized by short, slow strides and high step-to-step variability [2,3,4,5]. Gait changes associated with PD increase risk of falls [6] and significantly impact how individuals engage with the world around them. People with PD report that mobility impairments contribute to decreased quality of life, feelings of isolation, and fear of falling when performing activities [7,8,9]. Unfortunately, gait dysfunction is difficult to manage long-term with medication [10,11]; thus, allied health professionals require additional rehabilitative strategies to help foster safe and functional mobility in people with PD [12,13].

Rhythmic auditory stimulation cueing is a therapeutic alternative commonly recommended to regulate gait in people with PD [14,15,16]. Cueing capitalizes on our innate tendency to move to a beat in a synchronized manner by using auditory stimuli with regular, rhythmic properties to cue appropriate walking patterns [17,18]. Indeed, studies have shown that RAS^®^ and other cueing techniques improve gait velocity, cadence, stride length, double-limb support time, and gait variability in PD [19,20,21,22,23,24]. It is widely accepted that auditory cueing can benefit gait for individuals with PD; however, the exact effects of auditory cueing vary considerably in the literature [18,25,26,27,28,29,30] and clinicians lack concordant guidelines for how to appropriately implement auditory cueing for gait rehabilitation in PD.

Many factors are believed to influence gait responses to auditory cues, including the musical properties of auditory cues, individual factors, and instructional demands [31,32,33,34,35,36,37,38,39]. For example, some evidence suggests that synchronization is more accurate for familiar compared to unfamiliar music [32], especially among older adults with poor beat perception skills [39]. Familiarity with musical cueing stimuli may facilitate sensorimotor synchronization by reducing the cognitive demand associated with finding and walking to the beat [32]; however, the exact nature of this relationship is unclear, as other research has reported no effect of familiarity on gait during cueing [38]. Additionally, music that is higher in groove—or that elicits a strong desire to move [40]—increases stride velocity and length more than low-groove music [32,38,39,41] and may be as effective as a metronome at cueing appropriate gait [32,33,38,39]. Moreover, traits such as beat perception ability are also known to influence cueing outcomes, as poor beat perceivers typically perform worse than good beat perceivers during synchronized walking tasks [33,35,37]. This may be related to dual-task interference, as the cognitive demand associated with synchronizing to a beat while walking may be greater for individuals with poor beat perception abilities. Some evidence suggests that poor beat perceivers may benefit from altered synchronization instructions, as walking freely to music may be less cognitively demanding than consciously synchronizing to the beat [38].

Despite the growing body of research aimed at understanding factors that influence cueing efficacy in healthy adults, only a handful of studies have investigated the effects of music properties and beat perception ability on auditory cueing in clinical populations [34,35,42,43,44]. A recent study involving individuals with PD found that familiar music increases gait velocity and stride length from baseline to a greater extent than unfamiliar music [42]. Research has also suggested that rhythmic ability in people with PD may predict gait velocity changes during cueing [34]. Crucially, PD significantly impacts areas of the brain involved in various aspects of music processing [45,46], which may impose additional cognitive demand during music cueing therapy. Individuals with PD are also more susceptible to dual-task interference [47,48], which reduces gait speed, shortens steps, and increases variability despite exposure to auditory cues [49,50,51]. In general, rhythmic auditory cues or combined attentional and auditory cues improved parameters when measured in isolation, yet these benefits are inconsistent under dual-task challenges [49,50]. Therefore, research is needed to elucidate factors contributing to positive cueing outcomes and to explore how to mitigate the consequences of dual-task interference during cueing in people with PD.

The present study used an accelerated music-based auditory cueing paradigm to investigate the effects of stimulus familiarity, groove, beat perception ability, and synchronization instructions on gait outcomes in people with PD. To accomplish this, individuals with PD were randomized to instruction condition groups (free walking, synchronized walking) before walking to music excerpts played 10% faster than their baseline walking cadence. Music excerpts varied in familiarity (high, low) and perceived groove (high, low), and beat perception ability was assessed to highlight differences in gait responses between good and poor beat perceivers. Given the challenges associated with dual-tasking in PD, we predicted that poor beat perceivers would perform worse (e.g., wider strides, longer DLST, higher variability) when synchronizing to auditory cues, particularly during trials with unfamiliar music. Additionally, we expected high-groove music to elicit faster gait, higher cadence, and larger steps compared to low-groove music.

## 2. Method

### 2.1. Participants

23 volunteers diagnosed with idiopathic Parkinson’s Disease were recruited from the Southwestern Ontario community using community outreach and study flyers. Participants were eligible for the study if they could walk independently (i.e., without the aid of a person or mobility device), did not experience regular freezing of gait, and had been on a stable medication regimen for over four weeks; however, participants were not excluded based on medication (e.g., not currently taking medication), years since diagnosis, or previous treatments (e.g., deep brain stimulation) to facilitate the exploratory nature of this study. Thus, one individual not taking medication and one who had undergone deep brain stimulation were included. One participant was excluded due to technical errors and one due to cognitive difficulty with completing the full experiment. The final sample reported in the analyses includes 21 individuals. All participants provided written informed consent, as per the University of Western Ontario’s Human Research Ethics Board, and received monetary compensation for their time.

### 2.2. Procedures

Testing occurred during each participant’s self-reported peak “ON” phase of their medication cycle (approximately 45 min to one hour after taking medication). Participants completed a silent baseline walk followed by a rating task to identify stimuli for cued gait trials. Participants then completed two practice gait trials, followed by eight cued walking trials. Lastly, participants completed the Beat Alignment Test, a clinical assessment, and a demographics questionnaire.

#### 2.2.1. Clinical Evaluation and Demographics

To assess motor symptom severity and disease stage, the motor examination subsection of the Movement Disorder Society Unified Parkinson’s Disease Rating Scale (MDS-UPDRS III) [52] and the Timed Up-And-Go test (TUG) [53] were completed. These assessments were administered at the outset of the study, immediately prior to the experiment.

Participants also completed a demographic questionnaire and the Musical Training subscale of the GMSI [54]. To assess mental state for demographic purposes, participants completed the Montreal Cognitive Assessment version 7.2 (MoCA) [55], the Beck Depression Inventory (BDI) [56], the Beck Anxiety Inventory (BAI) [57], and the Starkstein Apathy Scale (SAS) [58].

#### 2.2.2. Baseline Gait Measurements

**Figure 1:**
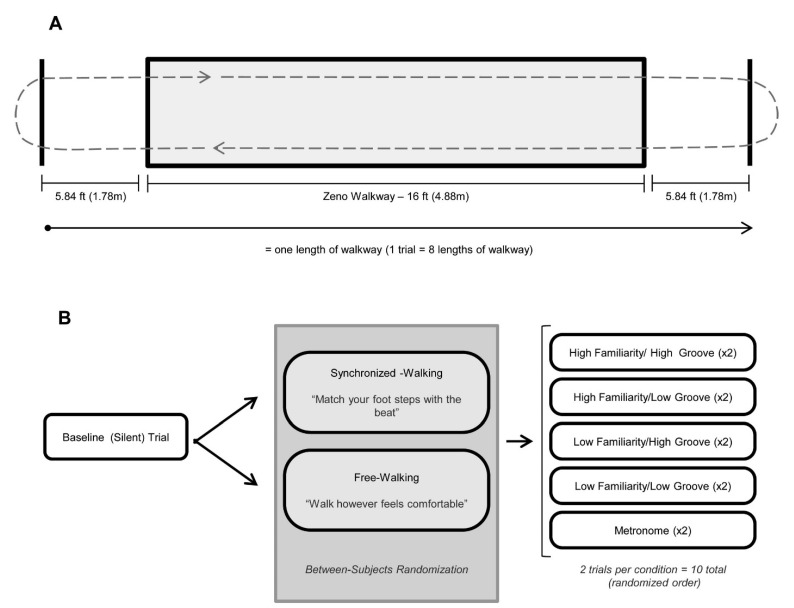
Gait procedures, adapted from Ready et al. [38,39]. (**A**): Illustration of pressure sensitive Zeno™ walkway procedures. All gait trials (baseline and cued) consisted of 8 consecutive passes of the 16 ft walkway (shaded gray rectangle). To reduce acceleration/deceleration effects, participants walked to a floor marking 1.78 m beyond the edge of the walkway (solid black lines) before turning and re-entering the walkway. (**B**): Protocol for gait trials. Gait was evaluated in silence (baseline–no cue) and during five randomly ordered cueing conditions: listening to music that was rated by the participant as (1) high familiarity/high groove [HFHG], (2) high familiarity/low groove [HFLG], (3) low familiarity/high groove [LFHG], (4) low familiarity/low groove [LFLG], and (5) a metronome. Two trials with distinct stimuli occurred for each condition except for metronome, which was identical in both trials. Participants were randomized to either synchronized walking (instructed to match their steps with the beat of the auditory cue) or free walking (instructed to walk however was comfortable, with the cue in the background).

To acquire baseline gait data, participants walked eight passes of a 4.88 m (16 ft) pressure sensitive walkway (Zeno™) in silence at a self-selected and comfortable walking pace (Figure 1A). Baseline data was obtained prior to presentation of auditory stimuli. To limit capture of acceleration/deceleration phases of gait and to ensure capture of steady-state walking, participants walked continuously between two floor markings 1.78 m beyond each end of the walkway until instructed to stop [59,60].

#### 2.2.3. Stimulus Selection

Each participant walked to an individualized list of stimuli chosen based on their ratings of familiarity and groove. To create the lists, participants listened to and rated selections from a piloted database of non-lyrical music clips (30 s each). To accommodate the constraints of testing participants during peak-on phase of their medication cycle, participants rated a subset of 20 songs from the database that had received consistent ratings from older adults in our previous work [39] (see Supplementary Material 1). All stimuli were digitally altered using Audacity^®^, version 2.3.1 (http://audacity.sourceforge.net accessed on 30 April 2019) to increase tempo (beats per minute) while preserving pitch. Our previous work reported that cueing at 15% faster than participants’ baseline walking pace shortened steps [39]; therefore, all stimuli in this study were adjusted to be 10% faster than baseline. Participants listened to adjusted music clips in a randomized order and rated each on familiarity, groove, enjoyment, and beat salience. All four ratings were made before moving onto the next clip. Enjoyment and beat salience ratings were included only as filler ratings and were not analyzed. Stimuli were presented over noise-canceling headphones (Bose^®^ Quiet Comfort 3) and were rated on a computerized 100 pt scale (see Supplementary Material 2). For each participant, a custom MATLAB script (R2016b) was used to select two stimuli based on familiarity and groove ratings for each of the following cueing conditions: high familiarity/high groove (HFHG), high familiarity/low groove (HFLG), low familiarity/high groove (LFHG), and low familiarity/low groove (LFLG). Finally, a metronome file (www.reztronics.com accessed on 13 May 2015) was adjusted to 10% faster than participants’ baseline walking cadence for use in two metronome-only trials.

#### 2.2.4. Cued Gait Trials

We used simple randomization (as implemented by a random number generation formula in Excel) to assign participants to one of the following instruction conditions: free walking or synchronized walking (Figure 1B). Free walkers were instructed to walk however felt most comfortable for them. In cases where participants queried if they should synchronize, they were instructed again to “walk however feels most comfortable.” Synchronized walkers were instructed to find the beat in the piece of music before beginning their walk and match their footsteps to the beat as best as possible. Walking on the spot prior to beginning was permitted. Synchronized walkers were instructed that the beat rate should be relatively similar to their silent walking rate and that they should not have to walk half of or double their normal walking rate to synchronize. All participants walked to stimuli that were 10% faster than their own baseline walking cadence.

Participants completed two practice trials to ensure understanding of the task. Participants then completed two gait trials for each of the five cueing conditions for a total of 10 trials (8 music trials, 2 metronome trials; Figure 1B). Cued trials followed the same protocol as baseline gait trials (Figure 1A). Participants listened to stimuli through wireless Sennheiser^®^ HDR 160 headphones, at a comfortable volume, to prevent the experimenter from hearing the music and inadvertently influencing the participant.

#### 2.2.5. Beat Alignment Test (BAT)

To quantify beat perception ability, participants completed the perception subtest of the Beat Alignment Test (BAT) from the Goldsmith Musical Sophistication Index v1.0 (GMSI) [61]. The GMSI provides a standardized score that represents musical training/ability based on normative data in the general Western population. Participants heard 17 music clips with a superimposed metronome beep track and judged if the beeps were aligned (on-beat) or misaligned (off-beat) with the beat in the music. Trials were randomized and participants were instructed to make judgements without moving to the music. Participants who scored below 65% accuracy were classified as poor beat perceivers and those who scored above 70% accuracy were classified as good beat perceivers. The discrete trial structure of the BAT (17 trials) means no participants could score between 65 and 70%. This cut-off is in line with previous literature using the BAT in auditory cueing studies [33,37,39] and with other means and medians from previously published BAT data from the Music and Neuroscience Lab [62].

#### 2.2.6. Data Analysis

Gait trials were processed using the ProtoKinetics Movement Analysis Software Package, version 5.06 (Protokinetics LLC, Havertown, PA, USA). The following dependent variables were analyzed: step length and stride width (spatial measures); cadence, stride velocity, and double-limb support time (DLST; temporal measures); and step length variability, step time variability, and stride velocity variability (variability measures; refer to Table 1 for definitions). DLST and stride width were also evaluated as indicators of stability [4].

Separate 4-way analyses of variance (ANOVAs) were run for each dependent variable, with familiarity (high, low) and groove (high, low) as within-subject variables and beat perception ability (good, poor) and instruction type (free walking, synchronized walking) as between-subject variables. Family-wise Bonferroni corrections were applied to the following families of tests to correct for multiple comparisons: spatial (step length, stride width), temporal (cadence, stride velocity, DLST), and variability (coefficient of variation for step length, for step time, and for stride velocity). Thus, alpha was corrected to 0.025 for spatial parameters, 0.017 for temporal parameters, and 0.017 for variability parameters.

When the assumption of sphericity was violated, the Greenhouse-Geisser correction was applied to adjust degrees of freedom. We also assessed the data for outliers and found one participant; however, their inclusion or exclusion did not change the pattern of results in any substantive way. Q–Q plots revealed mild deviations from normality in the residuals of stride width, CV stride time, and CV stride velocity. Given the lack of outlier influence and that ANOVA is generally robust to minor normality violations, and suitable non-parametric alternatives to mixed measures ANOVA are limited, we retained these analyses.

There were no significant effects of familiarity or beat perception ability on any dependent variable; therefore, analyses were collapsed across these factors and 2 × 3 ANOVAs (instruction type: free walking, synchronized walking; stimulus: metronome, low groove, high groove) are reported (see Supplementary Material 3 for original analyses). We also explored continuous relationships between both beat perception scores and familiarity ratings with gait outcomes. These analyses did not reveal stronger or more sensitive effects than the categorical approach reported. We present the binarized beat perception ability analysis because it is easier to interpret and the continuous analyses did not yield additional insights.

To account for individual differences (e.g., leg length, height), data were analyzed as normalized change scores:

$$\mathrm{normalized change score}=\frac{\mathrm{cued gait parameter}-\mathrm{baseline gait parameter}}{\mathrm{baseline gait parameter}}$$

To determine if cueing conditions or instructions altered gait from baseline, additional 2-way mixed-design ANOVAs (instruction type: free walking, synchronized walking; cueing condition: baseline [no cue], low groove, high groove, metronome) were conducted on the raw data for each dependent variable. Follow-up 1-way repeated measures ANOVAs were used to clarify the nature of significant interactions.

## 3. Results

A summary of demographic data for the two instruction groups is available in Table 2. A summary of raw descriptive statistics is available in Table 3.

### 3.1. Spatial Gait Parameters

#### 3.1.1. Step Length

**Figure 2:**
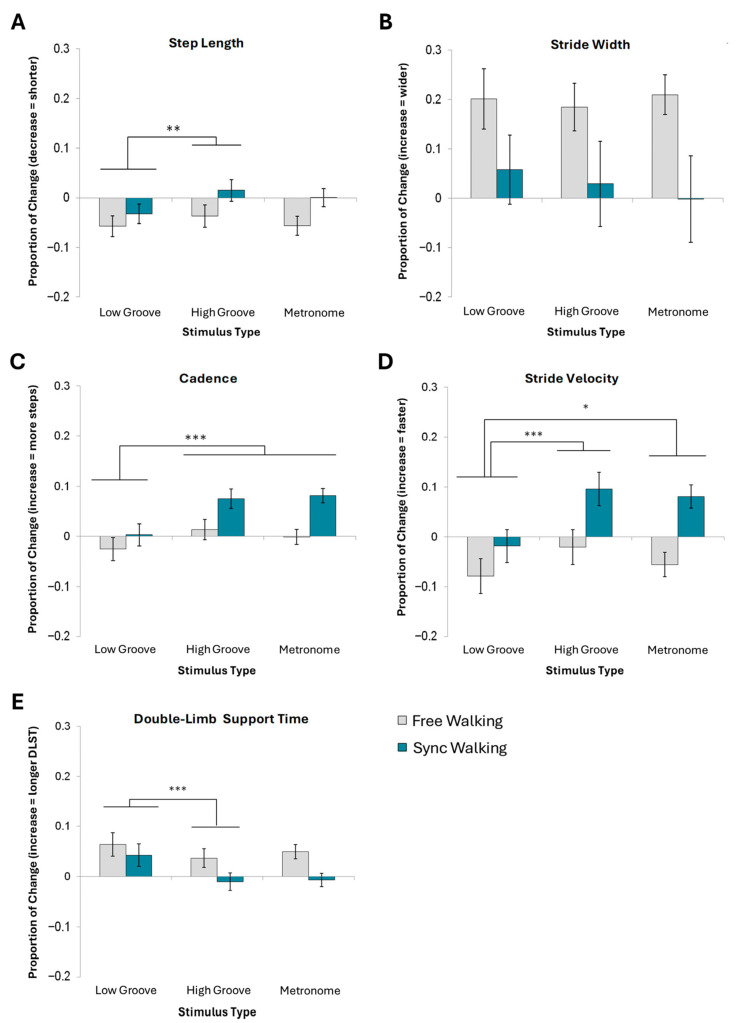
Mean normalized change scores and standard error for spatial and temporal gait measures. (**A**) Step length, (**B**) stride width, (**C**) cadence, (**D**) stride velocity, and (**E**) double-limb support time are shown. *** Denotes a main effect of stimulus type significant at *p* < 0.001. ** Denotes significance at *p* < 0.01. * Denotes significance at *p* < 0.05.

Overall, steps shortened from baseline. A 2 × 3 ANOVA indicated a significant main effect of stimulus on step length [*F*(1.8,34.7) = 5.19, *p* = 0.013, $\eta_{p}^{2}$ = 0.22]. Specifically, high-groove cues produced significantly larger steps [*M* = −0.011, *SD* = 0.07] than low-groove cues [*M* = −0.045, *SD* = 0.07], but not metronome cues [*M* = −0.028, *SD* = 0.07]. Step length did not significantly differ between metronome and low-groove cueing conditions (Figure 2A).

#### 3.1.2. Stride Width

A 4-way ANOVA revealed no significant effects of familiarity, groove, beat perception ability, or instructions on stride width (Figure 2B). Thus, no effects were collapsed for a 2 × 3 ANOVA (see Supplementary Material 3 for statistics).

### 3.2. Temporal Gait Parameters

#### 3.2.1. Cadence

A 2 × 3 ANOVA indicated a significant main effect of stimulus type on cadence [*F*(1.6,30) = 11.5, *p* < 0.001, $\eta_{p}^{2}$ = 0.38]. Participants took significantly more steps per minute to high-groove [*M* = 0.05, *SD* = 0.07] and metronome cues [*M* = 0.04, *SD* = 0.06] compared to low-groove cues [*M* = 0.01, *SD* = 0.07]. Cadence did not differ between metronome and high-groove cue conditions (Figure 2C). No significant effects of instruction were present following the multiple comparisons correction.

#### 3.2.2. Stride Velocity

A 2 × 3 ANOVA revealed significant main effects of stimulus type [*F*(1.7,32.2) = 11.30, *p* < 0.001, $\eta_{p}^{2}$ = 0.37] and instruction type [*F*(1,19) = 7.47, *p* = 0.013, $\eta_{p}^{2}$ = 0.28] on stride velocity. Stride velocity was significantly faster for both high-groove [*M* = 0.04, *SD* = 0.12] and metronome cues [*M* = 0.06, *SD* = 0.10] than for low-groove cues [*M* = −0.05, *SD* = 0.11]. Synchronized walking [*M* = 0.05, *SD* = 0.09] was associated with a faster velocity than free walking [*M* = −0.05, *SD* = 0.09] (Figure 2D).

#### 3.2.3. Double-Limb Support Time (DLST)

A 2 × 3 ANOVA indicated a significant main effect of stimulus type on DLST [*F*(1.5, 27.7) = 7.74, *p* < 0.01, $\eta_{p}^{2}$ = 0.29]. Specifically, high-groove cues [*M* = 0.01, *SD* = 0.06] yielded significantly lower DLST than both metronome [*M* = 0.02, *SD* = 0.05] and low-groove cues [*M* = 0.05, *SD* = 0.07]. Metronome cues elicited significantly less DLST than low-groove cues but significantly more DLST than high-groove cues (Figure 2E).

### 3.3. Variability Gait Parameters

**Figure 3:**
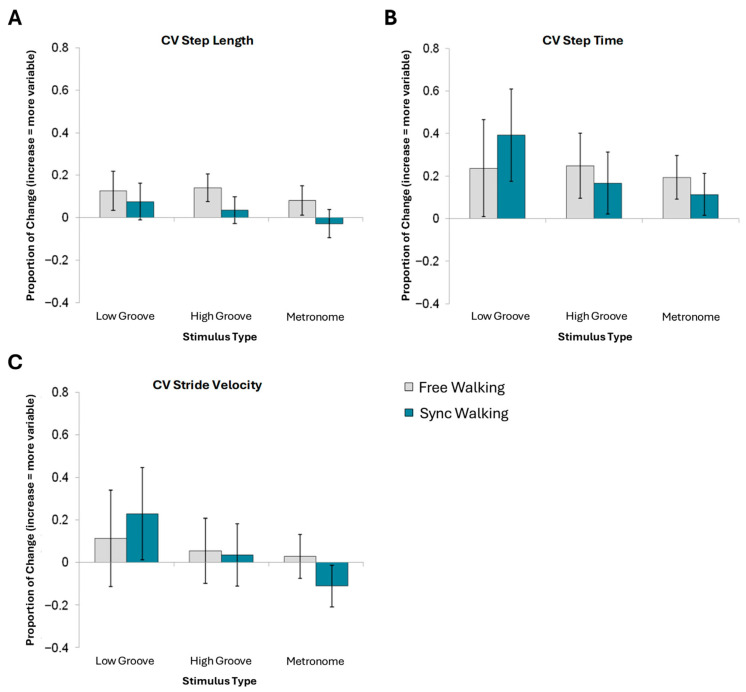
Mean normalized change scores and standard error for variability measures. No effects of stimulus type nor instruction reached significance for (**A**) CV step length, (**B**) CV step time, or (**C**) CV stride velocity.

There were no significant effects of any of the four factors on coefficients of variance for step length (Figure 3A), step time (Figure 3B), or stride velocity (Figure 3C). Therefore, these analyses were not rerun as 2 × 3 ANOVAs with instruction and stimulus type (see Supplementary Material 3 for statistics).

## 4. Discussion

The current study explored how gait in PD is influenced by familiarity, groove, beat perception ability, and synchronization demands during accelerated music-based rhythmic auditory cueing. Good and poor beat perceivers with PD were randomized to either walk freely with or synchronize to the beat of auditory cues—varying in familiarity and perceived groove—at a rate 10% faster than their baseline walking pace. As predicted, high-groove cues elicited faster gait speed, longer steps, higher cadence, and lower DLST than low-groove cues. Similarly, synchronized walking to auditory cues was associated with an overall higher velocity than free walking. Contrary to our hypothesis, instruction type did not appear to interact with beat perception ability, which may suggest that instructions to synchronize are not strongly associated with dual-task interference in poor beat perceivers with PD.

### 4.1. Groove Alters Gait in PD

High-groove music elicited faster overall gait speed, with lower DLST, higher cadence, and longer steps, than low-groove music. These results resemble previous findings in healthy young [32,33,38,39] and older adults [39], and support the notion that low-groove music does not produce the same beneficial gait outcomes as high-groove music. Importantly, we also found that low-groove cues increased DLST and decreased step length significantly from baseline. Low-groove cues were less effective than high-groove cues at normalizing gait and contributed to worse outcomes by shortening steps and negatively impacting gait stability. Overall, our results suggest that high- and low-groove music should not be used interchangeably during cueing therapy, and that high-groove music is preferential for improving gait.

### 4.2. Music- and Metronome-Based Auditory Cueing

High-groove cues and metronome stimuli did not produce different effects on any gait parameter in people with PD. This is in line with several studies suggesting that high-groove music and metronome cues yield similar outcomes on gait [32,33,38,39] and supports the conclusion from de Bruin et al. [20] that music is a viable alternative to metronome cues; however, we report that not all music is as effective as a metronome. Both high-groove and metronome stimuli increased velocity and cadence compared to low-groove music. Thus, groove should be considered when recommending and choosing auditory cues for interventions.

While our findings suggest that high-groove music can produce gait effects comparable to metronome cueing, the underlying neurological mechanisms may differ between these stimulus types. Metronome-based rhythmic auditory stimulation likely engages auditory-motor coupling pathways through the supplementary motor area and basal ganglia, or potentially lateral premotor and cerebellar ‘external cueing’ pathways that might be triggered by a metronome [65]. High-groove music may activate these same areas but may also recruit a broader network including reward-processing areas (ventral striatum, nucleus accumbens) [66]. This broader network engagement may explain why groove-based cueing can be equally effective despite its greater complexity as a cue—the enhanced motivation and emotional engagement triggered by high-groove music may compensate for any increased cognitive load through heightened attention and motor drive. In PD populations, where dopaminergic dysfunction affects both motor control and reward processing, high-groove music may provide alternative pathways for motor facilitation through preserved reward and emotional networks. This mechanistic distinction has important clinical implications, as it suggests that music-based interventions may be particularly beneficial for individuals with PD who show preserved emotional and reward processing despite motor impairments.

### 4.3. Synchronizing Enhances Auditory Cueing Outcomes in PD

This study demonstrated that synchronized walking is associated with greater increases in velocity and cadence than freely walking with music in the background. Little is currently known about the importance of synchronization instructions on cueing outcomes in PD. Our results indicate that synchronized walkers, but not free walkers, significantly increase gait velocity and cadence from baseline and suggest that instructions to synchronize may be crucial for entrainment in people with PD during auditory cueing therapy. Importantly, synchronized walking was not associated with deterioration in stability-related parameters (e.g., DLST, stride width) or parameters linked to higher fall risk (i.e., step length or step time variability); therefore, our findings provide evidence that attempting to synchronize to auditory cues does not negatively impact gait in people with PD and may instead improve therapy responses.

### 4.4. Beat Perception and Dual-Tasking

We hypothesized that synchronizing to the beat would worsen gait parameters in poor beat perceivers with PD, as finding and walking to the beat could increase cognitive demand and lead to dual-task interference. Our results did not support this idea, as we found no effect of beat perception on any outcome gait parameter. Previous studies have reported that rhythmic ability is linked to cueing outcomes in healthy [33,37,38] and clinical populations [34]; therefore, it is possible that an effect exists but was not captured in this study due to the relatively small sample size and correction for multiple comparisons. Our sample was also restricted to individuals who were able to ambulate safely without a mobility device, and, thus, may not have captured the full relationship between beat perception ability and dual-task interference in people with PD.

It is also possible that individuals with mild-to-moderate PD can overcome the effects of poor beat perception using other strategies. Ready et al. [38] suggested that people with worse rhythmic skills may rely on musical properties other than the beat when walking to music, which may explain why we found no effect of beat perception ability. A similar suggestion was made by Grahn and Brett [46], who stated that people with PD may not use beat structure as effectively to enhance performance on rhythm-based tasks, but that in paradigms involving real world music, including the present study, people with PD may mitigate beat perception impairments by using other acoustic information (e.g., changes in amplitude or regular percussion sounds) that contribute to musical properties like perceived groove. People with PD may even retain relatively preserved beat perception when embedded in musical contexts, even if they exhibit deficits in the context of rhythm alone. Music may also enhance motor timing via reward-related or emotionally salient mechanisms, thereby reducing reliance on precise rhythm perception. Another possibility is that poor beat perceivers are not consciously aware of their difficulties with beat finding or of discrepancies between the perceived and actual beat. As a result, their gait pattern is not negatively affected by synchronizing to a beat.

Furthermore, the absent effect of beat perception ability in the current study could also be influenced by cue pace. In clinical practice, auditory cues are typically presented 15% faster than an individual’s baseline walking cadence; however, some studies have found that cues 15–22.5% faster than baseline negatively impact gait in poor beat perceivers [32,33]. Similarly, our previous work [39] found that cueing at 15% faster than baseline cadence shortens steps in healthy young and older adults. To minimize this effect, the present study cued people with PD at 10% faster than baseline and found that poor beat perceivers performed similarly during free and synchronized walking. Given that other research cueing participants at baseline cadence also found no negative effects of beat perception ability on gait outcomes [38], it is possible that synchronizing is not as difficult when cues are closer to baseline walking pace and, thus, does not contribute to dual-task interference.

### 4.5. Accelerated Auditory Cues Do Not Increase Step Length

Overall, accelerated auditory cues appear to increase gait velocity and cadence in people with PD but do not increase stride length. Shortened strides are one of the primary gait changes in PD [67]; therefore, rehabilitative gait strategies should aim to normalize stride and step length alongside speed and stability. It is also important that researchers and clinicians consider the implications of cueing-induced gait changes within the context of more complex gait behaviors. For example, festination is a common gait pattern adopted to help recover displaced center of gravity through increasingly rapid and short steps [68,69,70]. If accelerated auditory cueing increases step rate and gait speed without altering step length, the risk of festination may also rise. The exact causes of gait festination are not well understood [70] and there is very little known about the effects of auditory cueing on festination (likely due in part to the safety risks associated with this research). To negate any chance that increasing gait speed without altering step length raises the risk of festination, future research should explore how accelerated auditory cues may be paired with additional strategies aimed at increasing step length.

Various studies have compared slower or preferred pace cues with faster cues to determine effectiveness (e.g., refs) [49,71,72,73,74,75]. In general, the rate of auditory cueing relative to a patient’s preferred cadence influences its effectiveness [18]. A recent meta-analysis [18] found that faster cueing yielded medium positive effects on velocity, small effects stride length, and large effects on cadence. In contrast, slower cueing yielded negative effects on velocity and cadence but small-to-medium positive effects on stride length. Although these findings align generally with our choice to implement a 10% faster cadence, it is important to recognize that individual responses may vary, and patients might benefit from slower cues if stride length is negatively affected by faster cues, especially in later disease stages where stability is of greater concern. 

### 4.6. Familiarity Did Not Significantly Affect Gait Measures

While prior studies have demonstrated that musical familiarity can enhance the efficacy of gait cueing—improving parameters such as stride length, velocity, and variability in people with PD [42,76], as well as cadence in healthy younger adults [32], we did not observe significant effects of cue familiarity in our cohort. This divergence may stem from several factors. First, prior studies demonstrating familiarity effects generally report small effects of familiarity, and we may have been underpowered to detect such effects. Second, prior studies may include repeated exposure [32,76], potentially increasing both familiarity and entrainment over time. In contrast, our assessment was acute and stimuli did not repeat. Third, our PD cohort may have differed in terms of cognitive load, motor severity, or baseline responsiveness to cueing, potentially moderating familiarity effects. Lastly, it is possible that when tempo and musical features are tightly controlled—as in our paradigm—the marginal gains from familiarity are attenuated. Our findings suggest that while familiarity may enhance cueing in some contexts, its influence is not particularly robust, and further research is needed to clarify the conditions under which familiarity modulates gait responses in PD.

### 4.7. Limitations

The exclusion of participants with frequent freezing of gait and those requiring mobility assistance limits the generalizability of our findings to the broader PD population, as these individuals may exhibit different responses to auditory cueing due to more severe motor symptoms and increased susceptibility to dual-task interference; however, meta-analytic evidence suggests that auditory cueing remains beneficial across disease stages, with slower-paced tempi (≥10% below preferred cadence) potentially offering particular advantages for individuals with advanced PD by promoting longer strides and more stable gait patterns despite reductions in walking speed [18].

The persistence and clinical meaningfulness of cueing-related gait improvements remain under investigation. Longitudinal studies have shown mixed results—some indicate that benefits diminish without continued training [77], while others report lasting improvements [78,79]. The individualized nature of our approach may enhance adherence and long-term engagement, as personally meaningful music appears more motivating and emotionally salient compared to generic metronomic cues [35,80]. Interestingly, familiarity may be more important for the enjoyment of music-related activities for people with PD than controls [81], thus, even if familiarity does not improve gait, it may enhance adherence. However, implementation challenges remain, including the need for ongoing music curation, potential habituation to repetitive stimuli, and variable technological proficiency among older adults with Parkinson’s disease [82]. Regarding clinical significance, meta-analyses and systematic reviews have suggested that music-based movement therapies are clinically meaningfully and improve motor function in PD [83], and that cueing significantly reduces the disability scores in the UPDRS-III motor subscale [29,77,84,85]. 

## 5. Conclusions

The purpose of this study was to explore how familiarity, groove, beat perception ability, and synchronization instructions influence gait outcomes in people with PD during accelerated cueing. High -groove music and synchronized walking increased velocity and cadence when individuals with PD walked to cues 10% faster than their baseline walking pace. Stimulus familiarity and beat perception ability had little effect on gait. We did not find any effects on gait variability measures. Overall, accelerated auditory cueing fostered faster gait patterns in individuals with PD. Further investigation is needed to determine how to best increase stride length in conjunction with gait speed for effective treatment of gait dysfunction in PD.

## Figures and Tables

**Table 1:** Definitions of gait parameters [63,64]. Adapted from Ready et al., 2022 [39].

Variable	Definition	Unit
Step Length	Distance from heel contact of one foot to the heel contact of the contralateral foot	cm
Stride Width	Distance between a line connecting the two ipsilateral foot heel contacts (the stride) and the contralateral foot heel contact between those events; measured perpendicular to the stride	cm
Cadence	Ratio of stride length by the stride time	cm/s
Stride Velocity	Steps per minute, obtained after dividing the number of footfalls minus one by the ambulation time	steps/min
Double-Limb Support Time	Number of seconds with both feet on the ground at the end of the stance phase, presented as a percentage of the gait cycle time	% sec
Step Length Variability	Coefficient of variation [average standard deviation in the gait parameter divided by the average mean; CV] of step length	%
Step Time Variability	CV of step time (where step time is the number of seconds from one step to the first contact on the following, contralateral foot; used as a proxy for cadence variability	%
Stride Velocity Variability	CV of stride velocity	%

**Table 2:** Demographic data by instruction and beat perception ability subgroup.

	Free Walking			Synchronized Walking		
	Poor BP (*n* = 6)	Good BP (*n* = 4)	All (*n* = 10)	Poor BP (*n* = 4)	Good BP (*n* = 7)	All (*n* = 11)
Age (years)	72.3 (1.6)	68.8 (10.8)	70.9 (6.7)	67.3 (11.1)	66 (7)	66.5 (8.2)
Sex (M/F)	5/1	2/2	7/3	2/2	6/1	8/3
MDS-Unified PD Rating Scale (Section III)	42.5 (15.1)	29 (15.2)	37.1 (16.0)	35 (16.9)	32.4 (16.0)	33.4 (15.5)
Hoehn and Yahr Score	2.4 (0.5)	2.3 (0.5)	2.3 (0.5)	2.3 (0.5)	2.1 (0.4)	2.2 (0.4)
Timed Up-and-Go Test	12.9 (1.4)	10 (1.3)	11.7 (2)	12.8 (1.7)	10.5 (0.5)	11.4 (1.5)
Montreal Cognitive Assessment 7.2	24.8 (4.6)	26.3 (1.4)	25.4 (3.5)	26 (1.7)	26.9 (2.2)	26.5 (2.5)
Beat Alignment Test (% Accuracy)	53.9 (4.4)	80.9 (10)	64.7 (15.4)	54.4 (8.8)	75.6 (6.3)	67.9 (12.7)
Beck Depression Inventory	11.6 (2.8)	9 (2.9)	10.3 (2.9)	13.3 (10.6)	11 (4.7)	11.8 (6.9)
Beck Anxiety Inventory	6 (4.7)	11.5 (8.7)	8.2 (7.6)	10.5 (4.7)	10 (8.8)	10.8 (7.3)
Starkstein Apathy Scale	15 (2.8)	12.3 (4.5)	13.9 (3.6)	15 (4.5)	10.4 (5.5)	12.1 (5.4)
Goldsmith Musical Sophistication Index *	14.5 (7.2)	25.3 (13.1)	18.8 (10.8)	17.3 (9)	17.9 (7.3)	17.6 (7.5)
Dance Training (years)	0 (0)	0.3 (0.5)	0.3 (0.3)	0 (0)	1.8 (4.5)	1.1 (3.6)

Note: Means and standard deviations are presented for all items except sex (reported as male/female). Participants were randomly assigned to Free Walking or Synchronized Conditions. * Goldsmith Music Sophistication Index represents a norm referenced score of music training (out of 49). MDS = Movement Disorder Society. There were no significant differences between Free Walking and Synchronized Groups on any of the demographics above (all *p*’s > 0.19).

**Table 3:** Raw descriptive statistics for gait parameters in the cueing and instruction conditions.

		Baseline	Low Groove	High Groove	Metronome
Step Length					
	Free Walking	58.2 (7.8)	55 (8.9) †	56.2 (9.3)	55 (9.3)
	Synchronized Walking	59.3 (7)	57.6 (8.9) †	60.3 (8.6)	59.3 (7.3)
Stride Width					
	Free Walking	7 (3.7)	8.1 (4) †	8 (4)	8.1 (4.1)
	Synchronized Walking	7.3 (2.2)	7.8 (3.7) †	7.4 (2.5)	7.2 (2)
Cadence					
	Free Walking	109 (9.6)	106.3 (11.4)	110.6 (13.1)	109 (12.2)
	Synchronized Walking	106.5 (5.7)	106.9 (11.9)	114.5 (9.6) *	115.1 (6.1) *
Stride Velocity					
	Free Walking	105.6 (18.9)	97.5 (20.8)	104 (24.2)	100.2 (23.4)
	Synchronized Walking	105.1 (13.6)	103.9 (21.7)	115.4 (19.6)	113.3 (13.8) *
Double Limb Support Time					
	Free Walking	17.1 (2.3)	18.2 (2.8) †	17.8 (2.9)	18 (2.8)
	Synchronized Walking	16.4 (1.5)	17.1 (2.8) †	16.3 (2.6)	16.3 (2.1)
CV Step Length					
	Free Walking	7.6 (2.9)	7.7 (2.7)	7.3 (2.8)	6.7 (2.2)
	Synchronized Walking	6.1 (3.4)	5.9 (2.9)	5.5 (2.7)	5.6 (1.8)
CV Step Time					
	Free Walking	4.7 (1.5)	4.7 (1.2)	4.6 (1.4)	3.8 (0.9)
	Synchronized Walking	5.4 (5)	4.4 (3.3)	4.1 (1.6)	3.7 (1.1)
CV Stride Velocity					
	Free Walking	5.7 (2.2)	5.4 (2.1)	5.3 (2.1)	5.2 (1.8)
	Synchronized Walking	6 (4.8)	5 (2.9)	4.2 (2)	4.7 (1.4)

Note: Raw means and standard deviations for each dependent variable averaged across beat perception group and familiarity. All reported effects are significant at the family-wise corrected alpha levels reported in the study methods. * Denotes significant change from baseline for stimulus type within an instruction group (stimulus type interacted with instruction). † Denotes significant change from baseline when averaged across instruction groups (stimulus type did not interact with instruction).

## Data Availability

The original contributions presented in this study are included in the article/Supplementary Material. Further inquiries can be directed to the corresponding author.

## Supplementary Materials

The following supporting information can be downloaded at: https://www.mdpi.com/article/10.3390/brainsci15090901/s1, Supplementary Material 1. Stimulus Database. Supplementary Material 2. End anchors for familiarity, groove, enjoyment, and beat salience ratings. Bold-faced text was not presented to participants. Supplementary Material 3. Results from original 2x2x2x2 ANOVAs showing effects of familiarity (high, low), groove (high, low), beat perception ability (good, poor), and instructions (free walking, synchronized walking) on spatial and temporal gait parameters.

## References

[1] Schapira A.H.V. (2009). Neurobiology and treatment of Parkinson’s disease. Trends Pharmacol. Sci..

[2] Bugalho P., Alves L., Miguel R. (2013). Gait dysfunction in Parkinson’s disease and normal pressure hydrocephalus: A comparative study. J. Neural Transm..

[3] Ebersbach G., Moreau C., Gandor F., Defebvre L., Devos D. (2013). Clinical syndromes: Parkinsonian gait. Mov. Disord..

[4] Hausdorff J.M., Cudkowicz M.E., Firtion R., Wei J.Y., Goldberger A.L. (1998). Gait variability and basal ganglia disorders: Stride-to-stride variations of gait cycle timing in Parkinson’s disease and Huntington’s disease. Mov. Disord..

[5] Švehlík M., Zwick E.B., Steinwender G., Linhart W.E., Schwingenschuh P., Katschnig P., Ott E., Enzinger C. (2009). Gait analysis in patients with Parkinson’s disease off dopaminergic therapy. Arch. Phys. Med. Rehabil..

[6] Schaafsma J.D., Giladi N., Balash Y., Bartels A.L., Gurevich T., Hausdorff J.M. (2003). Gait dynamics in Parkinson’s disease: Relationship to Parkinsonian features, falls and response to levodopa. J. Neurol. Sci..

[7] Marr J. (1991). The experience of living with Parkinson’s disease. J. Neurosci. Nurs..

[8] Schrag A., Jahanshahi M., Quinn N. (2000). How does Parkinson’s disease affect quality of life? A comparison with quality of life in the general population. Mov. Disord..

[9] Soundy A., Roskell C., Stubbs B. (2014). The experience of Parkinson’s disease: A systematic review and meta-ethnography. Sci. World J..

[10] Fahn S. (1999). Parkinson disease, the effect of levodopa, and the ELLDOPA trial. Arch. Neurol..

[11] Hung A.Y., Schwarzschild M.A. (2014). Treatment of Parkinson’s disease: What’s in the non-dopaminergic pipeline?. Neurotherapeutics.

[12] Deane K.H.O., Ellis-Hill C., Dekker K., Davies P., Clarke C.E. (2003). A Delphi survey of best practice occupational therapy for Parkinson’s disease in the United Kingdom. Br. J. Occup. Ther..

[13] Tomlinson C.L., Patel S., Meek C., Herd C.P., Clarke C.E., Stowe R., Shah L., Sackley C.M., Deane K.H.O., Wheatley K. (2012). Physiotherapy versus placebo or no intervention in Parkinson’s disease. Cochrane Database Syst. Rev..

[14] Aragon A., Kings J. (2018). Occupational Therapy for People with Parkinson’s. www.rcot.co.uk.

[15] Keus S.H.J., Bloem B.R., Hendriks E.J.M., Bredero-Cohen A.B., Munneke M. (2007). Evidence-based analysis of physical therapy in Parkinson’s disease with recommendations for practice and research. Mov. Disord..

[16] Sturkenboom I., Thijssen M., Gons-van Elsacker J., Jansen I., Maasdam A., Schulten M., Vijver-Visser D., Steultjens E., Bloem B., Munneke M. (2008). Guidelines for Occupational Therapy in Parkinson’s Disease Rehabilitation.

[17] Thaut M., Hoemberg V. (2014). Handbook of Neurologic Music Therapy.

[18] Ghai S., Ghai I., Schmitz G., Effenberg A.O. (2018). Effect of rhythmic auditory cueing on parkinsonian gait: A systematic review and meta-analysis. Sci. Rep..

[19] Brown L.A., de Bruin N., Doan J.B., Suchowersky O., Hu B. (2010). Obstacle crossing among people with Parkinson disease is influenced by concurrent music. J. Rehabil. Res. Dev..

[20] de Bruin N., Doan J.B., Turnbull G., Suchowersky O., Bonfield S., Hu B., Brown L.A. (2010). Walking with music is a safe and viable tool for gait training in Parkinson’s disease: The effect of a 13-week feasibility study on single and dual task walking. Park. Dis..

[21] McIntosh G.C., Brown S.H., Rice R.R., Thaut M.H. (1997). Rhythmic auditory-motor facilitation of gait patterns in patients with Parkinson’s disease. J. Neurol. Neurosurg. Psychiatry.

[22] Nieuwboer A., Kwakkel G., Rochester L., Jones D., van Wegen E., Willems A.M., Chavret F., Hetherington V., Baker K., Lim I. (2007). Cueing training in the home improves gait-related mobility in Parkinson’s disease: The RESCUE trial. J. Neurol. Neurosurg. Psychiatry.

[23] Rochester L., Hetherington V., Jones D., Nieuwboer A., Willems A.M., Kwakkel G., Van Wegen E. (2005). The effect of external rhythmic cues (auditory and visual) on walking during a functional task in homes of people with Parkinson’s disease. Arch. Phys. Med. Rehabil..

[24] Thaut M.H., McIntosh G.C., Rice R.R., Miller R.A., Rathbun J., Brault J.M. (1996). Rhythmic auditory stimulation in gait training for Parkinson’s disease patients. Mov. Disord..

[25] Lim I., van Wegen E., de Goede C., Deutekom M., Nieuwboer A., Willems A., Jones D., Rochester L., Kwakkel G. (2005). Effects of external rhythmical cueing on gait in patients with Parkinson’s disease: A systematic review. Clin. Rehabil..

[26] Spaulding S.J., Barber B., Colby M., Cormack B., Mick T., Jenkins M.E. (2013). Cueing and gait improvement among people with Parkinson’s disease: A meta-analysis. Arch. Phys. Med. Rehabil..

[27] Rocha P.A., Porfírio G.M., Ferraz H.B., Trevisani V.F. (2014). Effects of external cues on gait parameters of Parkinson’s disease patients: A systematic review. Clin. Neurol. Neurosurg..

[28] Forte R., Tocci N., De Vito G. (2021). The impact of exercise intervention with rhythmic auditory stimulation to improve gait and mobility in Parkinson disease: An umbrella review. Brain Sci..

[29] Ye X., Li L., He R., Jia Y., Poon W. (2022). Rhythmic auditory stimulation promotes gait recovery in Parkinson’s patients: A systematic review and meta-analysis. Front. Neurol..

[30] Harrison E.C., Earhart G.M. (2023). The effect of auditory cues on gait variability in people with Parkinson’s disease and older adults: A systematic review. Neurodegener. Dis. Manag..

[31] de Bruin N., Kempster C., Doucette A., Doan J.B., Hu B., Brown L.A. (2015). The effects of music salience on the gait performance of young adults. J. Music Ther..

[32] Leow L.A., Rinchon C., Grahn J.A. (2015). Familiarity with music increases walking speed in rhythmic auditory cuing. Ann. N. Y. Acad. Sci..

[33] Leow L.A., Parrott T., Grahn J.A. (2014). Individual differences in beat perception affect gait responses to low- and high-groove music. Front. Hum. Neurosci..

[34] Dalla Bella S., Benoit C.E., Farrugia N., Keller P.E., Obrig H., Mainka S., Kotz S.A. (2017). Gait improvement via rhythmic stimulation in Parkinson’s disease is linked to rhythmic skills. Sci. Rep..

[35] Dalla Bella S., Dotov D., Bardy B., Cochen de Cock V. (2018). Individualization of music-based rhythmic auditory cueing in Parkinson’s disease. Ann. N. Y. Acad. Sci..

[36] Leow L.A., Waclawik K., Grahn J.A. (2018). The role of attention and intention in synchronization to music: Effects on gait. Exp. Brain Res..

[37] Roberts B.S., Ready E.A., Grahn J.A. (2021). Musical enjoyment does not enhance walking speed in healthy adults during music-based auditory cueing. Gait Posture.

[38] Ready E.A., McGarry L.M., Rinchon C., Holmes J.D., Grahn J.A. (2019). Beat perception ability and instructions to synchronize influence gait when walking to music-based auditory cues. Gait Posture.

[39] Ready E.A., Holmes J.D., Grahn J.A. (2022). Gait in younger and older adults during rhythmic auditory stimulation is influenced by groove, familiarity, beat perception, and synchronization demands. Hum. Mov. Sci..

[40] Madison G. (2006). Experiencing groove induced by music: Consistency and phenomenology. Music Percept..

[41] Leow L.A., Watson S., Prete D., Waclawik K., Grahn J.A. (2021). How groove in music affects gait. Exp. Brain Res..

[42] Park K.S., Hass C.J., Janelle C.M. (2021). Familiarity with music influences stride amplitude and variability during rhythmically-cued walking in individuals with Parkinson’s disease. Gait Posture.

[43] Cochen De Cock V., Dotov D.G., Ihalainen P., Bégel V., Galtier F., Lebrun C., Picot M.C., Driss V., Landragin N., Geny C. (2018). Rhythmic abilities and musical training in Parkinson’s disease: Do they help?. npj Park. Dis..

[44] Patterson K.K., Wong J.S., Knorr S., Grahn J.A. (2018). Rhythm perception and production abilities and their relationship to gait after stroke. Arch. Phys. Med. Rehabil..

[45] Cameron D.J., Pickett K.A., Earhart G.M., Grahn J.A. (2016). The effect of dopaminergic medication on beat-based auditory timing in Parkinson’s disease. Front. Neurol..

[46] Grahn J.A., Brett M. (2009). Impairment of beat-based rhythm discrimination in Parkinson’s disease. Cortex.

[47] O’Shea S., Morris M.E., Iansek R. (2002). Dual task interference during gait in people with Parkinson disease: Effects of motor versus cognitive secondary tasks. Phys. Ther..

[48] Yogev G., Giladi N., Peretz C., Springer S., Simon E.S., Hausdorff J.M. (2005). Dual tasking, gait rhythmicity, and Parkinson’s disease: Which aspects of gait are attention demanding?. Eur. J. Neurosci..

[49] Lohnes C.A., Earhart G.M. (2011). The impact of attentional, auditory, and combined cues on walking during single and cognitive dual tasks in Parkinson disease. Gait Posture.

[50] Rochester L., Nieuwboer A., Baker K., Hetherington V., Willems A.-M., Chavret F., Kwakkel G., Van Wegen E., Lim I., Jones D. (2007). The attentional cost of external rhythmical cues and their impact on gait in Parkinson’s disease: Effect of cue modality and task complexity. J. Neural Transm..

[51] Baker K., Rochester L., Nieuwboer A. (2007). The immediate effect of attentional, auditory, and a combined cue strategy on gait during single and dual tasks in Parkinson’s disease. Arch. Phys. Med. Rehabil..

[52] Goetz C.G., Fahn S., Martinez-Martin P., Poewe W., Sampaio C., Stebbins G.T., Stern M.B., Tilley B.C., Dodel R., Dubois B. (2007). Movement Disorder Society-sponsored revision of the Unified Parkinson’s Disease Rating Scale (MDS-UPDRS): Process, format, and clinimetric testing plan. Mov. Disord..

[53] Podsiadlo D., Richardson S. (1991). The timed “Up & Go”: A test of basic functional mobility for frail elderly persons. J. Am. Geriatr. Soc..

[54] Müllensiefen D., Gingras B., Musil J., Stewart L. (2014). The musicality of non-musicians: An index for assessing musical sophistication in the general population. PLoS ONE.

[55] Nasreddine Z.S., Phillips N.A., Bedirian V., Charbonneau S., Whitehead V., Collin I., Cummings J.L., Chertkow H. (2005). The Montreal Cognitive Assessment, MoCA: A brief screening tool for mild cognitive impairment. J. Am. Geriatr. Soc..

[56] Beck A.T., Ward C.H., Mendelson M., Mock J., Erbaugh J. (1961). An inventory for measuring depression. Arch. Gen. Psychiatry.

[57] Beck A.T., Epstein N., Brown G., Steer R.A. (1988). An inventory for measuring clinical anxiety: Psychometric properties. J. Consult. Clin. Psychol..

[58] Starkstein S.E., Mayberg H.S., Preziosi T., Andrezejewski P., Leiguarda R., Robinson R.G. (1992). Reliability, validity, and clinical correlates of apathy in Parkinson’s disease. J. Neuropsychiatry Clin. Neurosci..

[59] Hollman J.H., Childs K.B., McNeil M.L., Mueller A.C., Quilter C.M., Youdas J.W. (2010). Number of strides required for reliable measurements of pace, rhythm and variability parameters of gait during normal and dual task walking in older individuals. Gait Posture.

[60] Rennie L., Löfgren N., Moe-Nilssen R., Opheim A., Dietrichs E., Franzén E. (2018). The reliability of gait variability measures for individuals with Parkinson’s disease and healthy older adults—The effect of gait speed. Gait Posture.

[61] Müllensiefen D., Gingras B., Stewart L., Musil J. (2014). The Goldsmiths Musical Sophistication Index (Gold-MSI): Technical Report and Documentation v1.0.

[62] Hsu P., Ready E.A., Grahn J.A., Kotz S. (2022). The effects of Parkinson’s disease, music training, and dance training on beat perception and production abilities. PLoS ONE.

[63] ProtoKinetics (2013). Measurements and Definitions.

[64] Winter D.A. (1991). Biomechanics and Motor Control of Human Gait: Normal, Elderly and Pathological.

[65] Nombela C., Hughes L.E., Owen A.M., Grahn J.A. (2013). Into the Groove: Can Rhythm Influence Parkinson’s Disease?. Neurosci. Biobehav. Rev..

[66] Matthews T.E., Witek M.A.G., Lund T., Vuust P., Penhune V.B. (2020). The Sensation of Groove Engages Motor and Reward Networks. Neuroimage.

[67] Morris M.E., Iansek R., Matyas T.A., Summers J.J. (1996). Stride length regulation in Parkinson’s disease: Normalization strategies and underlying mechanisms. Brain.

[68] Giladi N., Shabtai H., Rozenberg E., Shabtai E. (2001). Gait festination in Parkinson’s disease. Park. Relat. Disord..

[69] Morris M.E., Iansek R., Galna B. (2008). Gait festination and freezing in Parkinson’s disease: Pathogenesis and rehabilitation. Mov. Disord..

[70] Nonnekes J., Giladi N., Guha A., Fietzek U.M. (2019). Gait festination in parkinsonism: Introduction of two phenotypes. J. Neurol..

[71] Willems A.M., Nieuwboer A., Chavret F., Desloovere K., Dom R., Rochester L., Jones D., Kwakkel G., Van Wegen E. (2006). The use of rhythmic auditory cues to influence gait in patients with Parkinson’s disease, the differential effect for freezers and non-freezers, an explorative study. Disabil. Rehabil..

[72] Howe T.E., Lövgreen B., Cody F.W., Ashton V.J., Oldham J.A. (2003). Auditory cues can modify the gait of persons with early-stage Parkinson’s disease: A method for enhancing Parkinsonian walking performance?. Clin. Rehabil..

[73] Arias P., Cudeiro J. (2008). Effects of rhythmic sensory stimulation (auditory, visual) on gait in Parkinson’s disease patients. Exp. Brain Res..

[74] Picelli A., Camin M., Tinazzi M., Vangelista A., Cosentino A., Fiaschi A., Smania N. (2010). Three-dimensional motion analysis of the effects of auditory cueing on gait pattern in patients with Parkinson’s disease: A preliminary investigation. Neurol. Sci..

[75] Chester E.L., Turnbull G.I., Kozey J. (2006). The effect of auditory cues on gait at different stages of Parkinsonʼs disease and during “on”/”off” fluctuations. Top. Geriatr. Rehabil..

[76] Park K.S. (2022). Decomposing the effects of familiarity with music cues on stride length and variability in persons with Parkinson’s disease: On the role of covariates. Int. J. Environ. Res. Public Health.

[77] De Icco R., Tassorelli C., Berra E., Bolla M., Pacchetti C., Sandrini G. (2015). Acute and chronic effect of acoustic and visual cues on gait training in Parkinson’s disease: A randomized, controlled study. Park. Dis..

[78] Benoit C.-E., Dalla Bella S., Farrugia N., Obrig H., Mainka S., Kotz S.A. (2014). Musically cued gait-training improves both perceptual and motor timing in Parkinson’s disease. Front. Hum. Neurosci..

[79] Giorgi F., Donati D., Tedeschi R. (2024). Cueing interventions for gait and balance in Parkinson’s disease: A scoping review of current evidence. Appl. Sci..

[80] Brant M., Barrick C., Muno L., Stegemoller E. (2025). A pilot study on the influence of self-paced auditory cues and preferred music on gait in persons with Parkinson’s disease. Brain Sci..

[81] Morris I.B., Vasudevan E., Schedel M., Weymouth D., Loomis J., Pinkhasov T., Muratori L.M. (2019). Music to one’s ears: Familiarity and music engagement in people with Parkinson’s disease. Front. Neurosci..

[82] Devlin K., Alshaikh J.T., Pantelyat A. (2019). Music therapy and music-based interventions for movement disorders. Curr. Neurol. Neurosci. Rep..

[83] Zhou Z., Zhou R., Wei W., Luan R., Li K. (2021). Effects of music-based movement therapy on motor function, balance, gait, mental health, and quality of life for patients with Parkinson’s disease: A systematic review and meta-analysis. Clin. Rehabil..

[84] Calabrò R.S., Naro A., Filoni S., Pullia M., Billeri L., Tomasello P., Portaro S., Di Lorenzo G., Tomaino C., Bramanti P. (2019). Walking to your right music: A randomized controlled trial on the novel use of treadmill plus music in Parkinson’s disease. J. Neuroeng. Rehabil..

[85] Song J.H., Zhou P.Y., Cao Z.H., Ding Z.G., Chen H.X., Zhang G.B. (2015). Rhythmic auditory stimulation with visual stimuli on motor and balance function of patients with Parkinson’s disease. Eur. Rev. Med. Pharmacol. Sci..

