# Supplementary material for: Optimizing Gait Outcomes in Parkinson’s Disease: The Effects of Musical Groove and Familiarity
Source: Brain Sci. 2025 Aug 22;15(9):901. doi: 10.3390/brainsci15090901 (PMC12467459; doi:10.3390/brainsci15090901)
Supplement: Supplementary file 1 [file brainsci-15-00901-s001.zip › brainsci-3575873-supplementary.pdf]

Supplementary Material 1.
Stimulus Database.

Song Title	Familiarity	Groove
Copacabana	High	High
In the Mood	High	High
Green Onions	High	High
Twist and Shout	High	High
William Tell Overture	High	High
Something	High	Low
Nadia Theme	High	Low
Imagine	High	Low
Scarborough Fair	High	Low
Exodus	High	Low
Candy Rock	Low	High
Flip Flop	Low	High
Peach Fuzz	Low	High
Once More	Low	High
Cripple Creek	Low	High
Roses in December	Low	Low
White Keys	Low	Low
Albatross	Low	Low
To Audrey	Low	Low
Lullaby	Low	Low

Supplementary Material 2.
End anchors for familiarity, groove, enjoyment, and beat salience ratings. Bold-faced text was not presented to participants.

Familiarity: "How familiar is the piece of music to you?"	
1 = Never heard it before	100 = Know this song so well that I can predict what happens next
Groove: "How much does this piece of music make you want to move?"	
1 = Would definitely not move to this	100 = Would move a lot to this
Enjoyment: "How much do you enjoy listening to this piece of music?"	
1 = Strongly dislike this song	100 = Strongly enjoy this song
Beat Salience: "How strong is the beat in this piece of music to you?"	
1 = Very weak	100 = Very strong

Supplementary Material 3.
Results from original 2x2x2x2 ANOVAs showing effects of familiarity (high, low), groove (high, low), beat perception ability (good, poor), and instructions (free walking, synchronized walking) on spatial and temporal gait parameters.

	Step Length (cm)			Stride Width (cm)			Cadence (steps/min)			Stride Velocity (cm/sec)			DLST (sec)		
	F	p	η_p^2	F	p	η_p^2	F	p	η_p^2	F	p	η_p^2	F	p	η_p^2
Familiarity	0.17	0.69	0.01	2.31	0.15	0.12	0.11	0.75	0.01	0.06	0.81	0.00	0.56	0.47	0.03

Familiarity*Instruction	0.24	0.63	0.01	1.55	0.23	0.08	0.19	0.67	0.01	0.18	0.67	0.01	0.47	0.50	0.03
Familiarity*BP	1.51	0.24	0.08	2.86	0.11	0.14	1.81	0.20	0.10	2.16	0.16	0.11	1.18	0.29	0.06
Familiarity*Instruction*BP	0.99	0.33	0.05	1.12	0.31	0.06	1.34	0.26	0.07	1.68	0.21	0.09	0.88	0.36	0.05
Groove	11.9	0.00	0.41	0.61	0.45	0.03	21.96	0.00	0.56	24.07	0.00	0.59	24.20	0.00	0.59
Groove*Instruction	1.51	0.24	0.08	0.08	0.78	0.00	1.83	0.19	0.10	2.17	0.16	0.11	2.02	0.17	0.11
Groove*BP	1.12	0.31	0.06	0.13	0.72	0.01	0.05	0.83	0.00	0.50	0.49	0.03	0.59	0.45	0.03
Groove*Instruction*BP	2.64	0.12	0.13	0.04	0.85	0.00	0.03	0.87	0.00	0.62	0.44	0.04	1.29	0.27	0.07
Familiarity*Groove	0.68	0.42	0.04	1.82	0.20	0.10	0.57	0.46	0.03	1.05	0.32	0.06	0.00	0.95	0.00
Familiarity*Groove*Instruction	1.58	0.23	0.09	0.07	0.80	0.00	0.05	0.83	0.00	0.51	0.49	0.03	3.10	0.10	0.15
Familiarity*Groove*BP	0.04	0.85	0.00	0.63	0.44	0.04	6.33	0.02	0.27	2.06	0.17	0.11	0.25	0.62	0.01
Familiarity*Groove*Instruction*BP	0.35	0.56	0.02	0.43	0.52	0.02	2.06	0.17	0.11	0.36	0.56	0.02	0.00	0.98	0.00
Instruction	1.26	0.28	0.07	2.31	0.15	0.12	2.73	0.12	0.14	3.10	0.10	0.15	1.36	0.26	0.07
BP	0.56	0.47	0.03	0.03	0.88	0.00	0.18	0.68	0.01	0.10	0.75	0.01	0.00	1.00	0.00
Instruction*BP	0.63	0.44	0.04	0.23	0.64	0.01	0.29	0.60	0.02	0.41	0.53	0.02	0.68	0.42	0.04

Note. Bonferroni alpha adjustments were applied to adjust for multiple comparisons. Thus, the critical alpha value is 0.025 for spatial measures (step length and width) and is 0.017 for all temporal measures (cadence, velocity, DLST). DLST = double limb support time. BP = beat perception. η_p^2 = partial eta squared (effect *size*).

Results from original 2x2x2x2 ANOVAs showing effects of familiarity (high, low), groove (high, low), beat perception ability (good, poor), and instructions (free walking, synchronized walking) on CV of step length, time, and velocity.

	Step Length Variability (CV)			Step Time Variability (CV)			Stride Velocity Variability (CV)		
	F	p	η_p^2	F	p	η_p^2	F	p	η_p^2
Familiarity	5.02	0.04	0.23	0.51	0.49	0.03	1.22	0.28	0.07
Familiarity*Instruction	0.00	0.98	0.00	0.06	0.81	0.00	0.36	0.56	0.02
Familiarity*BP	0.82	0.38	0.05	0.64	0.44	0.04	0.09	0.77	0.01

Familiarity*Instruction*BP	4.65	0.05	0.21	1.12	0.31	0.06	3.11	0.10	0.15
Groove	0.09	0.77	0.01	1.26	0.28	0.07	3.13	0.09	0.16
Groove*Instruction	0.40	0.53	0.02	2.21	0.16	0.12	0.70	0.41	0.04
Groove*BP	0.00	0.96	0.00	0.04	0.85	0.00	0.33	0.57	0.02
Groove*Instruction*BP	0.00	0.99	0.00	1.65	0.22	0.09	0.00	0.99	0.00
Familiarity*Groove	2.03	0.17	0.11	0.95	0.34	0.05	0.05	0.82	0.00
Familiarity*Groove*Instruction	0.65	0.43	0.04	0.00	0.98	0.00	0.12	0.74	0.01
Familiarity*Groove*BP	0.29	0.60	0.02	0.67	0.43	0.04	0.00	0.95	0.00
Familiarity*Groove*Instruction*BP	4.47	0.05	0.21	0.79	0.39	0.04	0.48	0.50	0.03
Instruction	0.42	0.53	0.02	0.01	0.91	0.00	0.00	0.95	0.00
BP	0.09	0.77	0.01	0.00	1.00	0.00	0.69	0.42	0.04
Instruction*BP	0.04	0.84	0.00	0.15	0.71	0.01	0.25	0.62	0.01

Note. Bonferroni alpha adjustments were applied to adjust for multiple comparisons. Thus, the critical alpha for variability measures is 0.017. BP = beat perception. η_p^2 = partial eta squared (effect *size*).
